# The Effects of a Mandibular Overdenture on Edentulous Patients’ Quality of Life: A Clinical Study

**DOI:** 10.3390/healthcare11111577

**Published:** 2023-05-27

**Authors:** Edoardo Rella, Paolo De Angelis, Laura Papetti, Giovanni Damis, Antonio D’Addona, Paolo Francesco Manicone

**Affiliations:** Division of Oral Surgery and Implantology, Department of Head and Neck and Sensory Organs, Fondazione Policlinico Universitario A. Gemelli IRCCS—Università Cattolica del Sacro Cuore, 00168 Rome, Italy

**Keywords:** implant-supported prostheses, quality of life, overdenture

## Abstract

Edentulous patients’ quality of life can be greatly diminished by the use of a badly fitting removable prosthesis, as many aspects of social life become notably impaired. The object of this study was to evaluate if treating these patients with a two implants mandibular overdenture could improve their quality of life as measured with the Italian version of the OHIP-14 (Oral Health Impact Profile). Edentulous patients, in good clinical condition, were selected. Two implants were placed following the recommended guidelines and three months after new mandibular dentures were manufactured, implants were uncovered and connected to the prosthesis using LOCATOR abutments. OHIP-14 was measured at baseline, one month after delivery and one year after delivery. An improvement was observed even after one month (with a mean reduction of 17 points in OHIP) and that improvement appeared to be stable at the one-year follow-up. Mandibular overdentures can improve a patient’s quality of life when compared to a tissue-supported removable complete denture, as long as the patient is subjected to an appropriate follow-up as the retentive rings of the attachment can deteriorate even after two years and lose a great deal of retentive capabilities.

## 1. Introduction

Despite recent advances in dentistry, millions of people around the world, mainly because of caries and periodontal disease, end up edentulous, having to resort to prosthodontics. Edentulism greatly affects a patient’s quality of life, as it limits his ability to perform two essential tasks in life: speaking and eating. Therefore, those patients have to resort to different changes in order to compensate for their deficiencies [1,2].

Several treatments for edentulism are available, including, but not limited to, conventional removable complete dentures, implant-retained overdentures (IODs) and, in some cases, implant supported, full arch fixed restorations.

In the past, removable dentures have been the most common treatment option for edentulism, with different results in terms of patients’ satisfaction [3,4]. Furthermore, as a consequence of the bone resorption processes that normally happen in edentulous patients, and that are expedited by the use of removable complete dentures, their use is limited and might even be detrimental in the long run [5,6].

IODs represent an efficient option for treating edentulous patients, especially in the mandible, with the adoption of two unsplinted implants [7]; in fact, mandibular IODs have many advantages when compared to removable complete dentures. One of the primary benefits of implant-retained overdentures is the improved stability and retention compared to conventional dentures; another benefit of implant-retained overdentures is the preservation of bone and soft tissue. Dental implants integrate with the surrounding bone tissue, which helps to prevent bone loss and maintain the natural shape of the jaw as the ridge resorption process is notably slower, therefore granting an improvement of these patients’ quality of life [8,9]. Similarly, IODs can provide optimal support to the perioral tissues, improving the aesthetic of the perioral by restoring the lost vertical dimension [10,11].

To achieve satisfactory retention for edentulous patients at a reasonable cost, it is recommended by the McGill Consensus Statement that overdentures should be retained by two implants in the anterior area of the mandible [7]. The York Consensus Statement also supports the use of at least two implants to enhance retention, while the EAO consensus conference indicated that oral health-related quality of life can be improved by overdentures retained by two or four implants [12,13].

The costs of overdentures retained by two implants are somewhat similar to those of conventional complete dentures. Using unsplinted anchors can lower the cost of implant-retained overdentures compared to those with splinted anchors [14,15,16]. Even single implant overdentures can be efficient in improving patients’ quality of life, as demonstrated by Abdelal et al. [17]. Although, many articles have reported that single implant overdentures have a much higher rate of complications, starting from a significant high risk of prosthesis fracture [18].

Additionally, unsplinted mandibular implant-retained overdentures have a low repair frequency, are easy to maintain on isolated attachments, have a good emergence profile, and require simple oral hygiene practices in daily life [14]. Therefore, unsplinted mandibular implant-retained overdentures with at least two implants are frequently used in dental clinics [19,20].

The way a patient feels about their prosthesis is very important and should be incorporated into every treatment protocol. Unfortunately, patient’s comfort with their prosthesis is based on parameters that are totally different to clinical ones; patients usually rate their dentures on the basis of their ability to chew certain harder or softer solid foods, and to pronounce certain words.

Oral health is a critical component of overall health and well-being. Poor oral health can have negative impacts on an individual’s quality of life, including pain, discomfort, and difficulty eating and speaking [21,22]. Therefore, it is essential to measure and assess oral health-related quality of life (OHRQoL) to better understand the impact of oral health on individuals’ lives.

One commonly used tool to assess OHRQoL and patients’ satisfaction is the Oral Health Impact Profile (OHIP) [23]. The OHIP is a self-reported questionnaire that consists of 49 items that cover 7 dimensions: functional limitation, physical pain, psychological discomfort, physical disability, psychological disability, social disability, and handicap.

The OHIP has been widely used in research and clinical settings to assess OHRQoL in various populations, including children, adolescents, adults, older adults, and even on mandibulectomies patents [24,25,26].

Many shorter versions of this instrument are available, such as the OHIP (Oral Health Impact Profile)-14, OHIP-20, and the OHIP-EDENT, which are also considered valuable instruments, present a more succinct battery of questions, have been experimented in edentulous patients, and are capable of properly evaluating the perceived impact of oral health on subjects’ well-being [27,28].

The main purpose of this study is to evaluate the improvement in the perceived quality of life in edentulous patients after placing two implants and going from a removable complete mandibular denture to an implant-supported overdenture supported by two unsplinted implants. The null hypothesis is that a mandibular IOD would not improve the OHIP of edentulous patients previously treated with mandibular removable complete dentures.

## 2. Materials and Methods

The present study was conducted at a private practice (Rome, Italy) where all specialties of dentistry are commonly practiced. Patients who were treated with mandibular IODs, as they were not comfortable with their removable complete denture, were recruited. All procedures were performed according to the Declaration of Helsinki on experimentation involving human subjects. Due to the nature of the present study, it was granted an exemption, in writing, by the institutional review board. No additional intervention was delivered to those patients as a consequence of their recruitment, and they were subjected to a standard treatment plan.

Each participant who enrolled in the study received explanations about the study design and objectives and provided written informed consent.

### 2.1. Selecting Patients

Edentulous patients that were scheduled for a mandibular overdenture were included in this study. Patients with poor general health, with a severe systemic disease that is a constant life threat to the patient and that needs constant medical treatment (ASA 4) (the American Society of Anesthesiologists (ASA) physical status classification), moribund patients (ASA 5), or patients that suffered from diseases or were under medications (e.g., Bisphosphonates) that constituted a contra-indication to an implant-supported rehabilitations were excluded. Similarly, patients that received head-and-neck radiotherapy or had undergone a surgical procedure for treating a head-and-neck tumor were excluded from the present study.

### 2.2. Surgical Procedure

A preoperative radiographical evaluation was conducted on each patient to evaluate the residual bone volumes and the most appropriate location for each implant. Implants were planned following modern guidelines [29,30].

The same experienced (20+ years of placing implants) operator performed all the surgeries. Before implant placement, all patients received 2 mg of Amoxicillin + Clavulanic Acid (Augmentin, GlaxoSmithKline, Brentford, UK); a mandibular full-thickness flap was raised, and two implants from multiple implant brands (Zimmer Biomet, Warsaw, IN, USA or Straumann, Basel, Switzerland) were inserted in the canine/lateral incisor position in each participant, according to the drilling protocol recommended by the manufacturer, under local anesthesia obtained with articaine 2% with 1:100,000 epinephrine (Articaina 2%, OGNA, Milan, Italy). A cover screw was placed, and the flap was sutured using 5-0 nylon (Monosoft, Syneture, Covidien, Dublin, Ireland) interrupted sutures.

After implant placement, patients received oral and written recommendations on medication, oral hygiene, prosthesis maintenance, and diet. Analgesics were administered as needed. Patients were not allowed to wear the denture for 14 days. Sutures were removed 2 weeks after the surgery.

### 2.3. Prosthetic Rehabilitation

Three months after initial surgery, patients received a new mandibular removable complete denture following standardized techniques:

1. First appointment: An alginate impression was taken using a tray optimal for edentulous patients.
2. Second appointment: A second impression was taken with an individual tray using a polysulfide impression material (Permlastic, Kavo Kerr, Romulus, MI, USA), obtaining optimal mucosal support and extension.
3. Third appointment: The vertical dimension of occlusion was registered.
4. Fourth appointment: The prosthesis was tried, adopting a lingualized occlusion scheme, free from any premature contact [31].
5. Fifth appointment: The prosthesis was delivered removing any area of excessive pressure.

At the delivery appointment, a second full-thickness flap was then raised, implants were uncovered, and LOCATOR abutments (Zest Dental Solutions, Carlsbad, CA, USA) were placed over the two implants. Each LOCATOR abutment was chosen on the basis of the available soft tissues. Using a calibrated periodontal probe, the thickness of supra-crestal tissues was measured and a LOCATOR of height equal to the thickness + 1 mm was chosen, following the manufacturer’s instructions. At the same appointment, the denture was connected to the abutments. The housings were placed on the implants engaging the connections over the abutments, dentures were relieved in the area that was to harbor the housings, acrylic resin was then placed in those areas and over the housings and the prosthesis was positioned and kept in place by inviting the patients to bite down. Once the resin had set, the IODs were finished extra orally, and retention inserts of medium strength were positioned; all prosthesis had at least a high retention. Patients’ occlusion was checked removing any precontact and allowing for a balanced occlusion scheme, and the prostheses were delivered after teaching each patient how to properly remove and insert their IOD.

### 2.4. Follow-Up and Data Recording

Patients were then recalled 1 month (T1) and 1 year (T2) after implant loading; after removing the IODs, locator abutments were examined visually for signs of wear such as any fissures or break lines. The abutment was also surveyed for stability. Each housing was also checked to observe any sign of deterioration of the two inserts.

Each IOD was inserted and removed by an investigator to appraise the retention force of the attachment and describe it on a subjective scale from 1 to 5 (1 = very high; 2 = high; 3 = average; 4 = weak; 5 = very weak). If the value was 4 or 5, both Locator male parts were changed. A single examiner oversaw these evaluations to reduce any risk of bias. Patients were also recalled after two years (T3); at that appointment prosthesis retention was also measured.

This examiner was previously calibrated, measuring the retention force of the same 20 prosthesis twice at a one-week interval, and a Cohen’s kappa of 0.87 was recorded.

### 2.5. OHIP Measurement

Oral health-related quality of life was evaluated before implant treatment (T0), 1 month (T1) and 1 year after implant loading (T2), using the Italian version of the OHIP-14. The adopted questionnaire is presented in Appendix A. The questionnaire was obtained by translating the English version and then by back translating the questionnaire into English, checking for any difference between the original questionnaire and the obtained version. Two authors (E.R. and P.D.A.) oversaw this process.

The OHIP-14 scale consists of 14 items that investigate consequences of oral-related disorders.

The responses are made on a 5-point Likert type scale: 0 = never, 1 = hardly ever, 2 = occasionally, 3 = often, and 4 = very often.

Summary scores were calculated considering all 14 OHIP items, obtaining a final score (range 0–56) adopted as the main outcome.

### 2.6. Data Analysis

All collected data were analyzed and reported as mean ± standard deviation for numerical variables, categorical variables were reported as absolute and relative frequencies.

The main outcome was the comparison of OHIP-14 values at T0 and T1 and T2, to provide an overview of whether the application of an implant-supported prosthesis had any positive effect on the oral-related quality of life of these patients, and if these improvements, if any, were stable after one year.

The secondary outcome was to measure the retention force provided by the attachments and to provide an overview of any diminishment of this force one year and two years after delivery of the prosthesis.

After having checked for normality using the Shapiro–Wilk test, the values obtained at the three time points were compared using the analysis of variance (ANOVA) test for repeated measurements. Bonferroni correction was used for post-hoc comparisons. *t*-test for repeated measurements was used to compare the retention values measured at T2 and at T3.

## 3. Results

From an initial pool of 53 eligible patients (Table 1), 5 patients moved out of the city and 8 patients were not available or did not want to participate in the cross-sectional study, leaving a total of 40 participants; 42.5% of the subjects (n = 17) were men, while 57.5% of them (n = 23) were women, with a mean age of 65.48 ± 5.23. All implants were still in use at the end of the follow-up, but 4 LOCATOR abutments in 3 patients had to be retightened as patients claimed discomfort during function.

The OHIP analysis that was conducted on 40 patients showed a significant advancement comparing the preoperative status and the status one year after prosthetic loading as the OHIP-14 summary value went from 37.27 ± 3.49 at T0, to 20.3 ± 4.17 at T1, while the summary value, as measured at the one-year follow-up was 20.675 ± 3.89. (Table 2 and Table 3).

The repeated measures ANOVA showed a significant difference between the overall score at the three time points (F-value = 459.00; *p* < 0.001). The extremely high F-value shows that the difference between groups is much higher than the difference within groups. The post-hoc pairwise *t*-tests adopting Bonferroni’s correction showed a statistically significant difference between the overall values as measured at T0 and those measured at T1 and T2; no statistically significant difference was found comparing values measured at T1 and at T2.

When analyzing the efficiency and retention of the LOCATOR attachments, at T2 a mean value of 2.22 ± 0.69 was found, while at T3 a mean value of 3.15 ± 0.89 was recorded, showing a significant difference between the two time points; most importantly, at T2 no values higher than three were recorded, while at T3 no values lower than two were recorded and half the sample had a retention deemed as average, with four subjects showing a very weak retention (Table 4).

## 4. Discussion

This study aims to observe the efficacy of mandibular IODs supported by two implants in improving patients’ quality of life at different follow-ups.

It has been reported that satisfaction is the most important outcome for edentulous patients [32]; therefore, it should be measured properly to better adapt our rehabilitations. As Locker said in 1998, the ultimate aim of any intervention should be to reduce pain and discomfort and to improve function and social psychosocial well-being [33]. Patients rehabilitated with IODs showed an immediate improvement of their quality of life, as expected given the higher retention offered by implants, and as it clearly appears from many other studies available in the literature [34,35,36]. Our paper applies the OHIP 14 in edentulous patients at a long follow-up, and simultaneously investigates the retention offered by the prostheses. Furthermore, we repeated those measurements in order to not only investigate the immediate effects of the overdentures, but also their stability over time—an analysis which is not so common in the literature.

In our study, after receiving their IOD, patients showed an improvement in all 14 items that compose the OHIP-14, with the highest difference being in the Q4, Q11, and Q14 items. Therefore, IODs can not only improve patients’ capability of eating but can offer an overall significant improvement in these patients’ lives as they found themselves being less angry and irritable than before, and capable of enjoying more activities that they had once found precluded to them.

The null hypothesis was therefore rejected: the quality of life of patients treated with mandibular overdentures did indeed improve.

Not only that, but our study also shows that mandibular IODs can provide a stable result in these patients even after a long follow-up (two years). This is a finding of great relevance; IODs have the higher rate of complications among all implant-retained restorations and the wear of the retaining elements of the prosthesis could quickly lead to a decrease in patients’ satisfaction [18,37].

Regarding the retentive capabilities of these prostheses, many studies have shown that bar-supported IODs provide, by far, the greatest retention [38]. Similarly, LOCATOR attachment provides the highest satisfaction, while ball attachment, even if they do not offer any particularly superior capability in regard to the other two retentive systems, can still be of great use in cases of a reduced vertical dimension [39].

In our study, only LOCATOR abutments were adopted, given their efficacy in treating edentulous patients; after one year, no prostheses had a retention worse than moderate, showing that LOCATOR abutments can offer enough retention after one year of use. After two years still more than half of prostheses (72.5%) had a retention value lower than 3 and only 11 prostheses (28.5%) had a weak or very weak retention. We therefore suggest that these patients should be recalled after two years to inspect both the abutments and the retentive ring to observe if the retention provided is enough and, therefore, change those retentive rings if needed.

Our results are comparable to what other studies have shown. Many studies have shown that IODs, when compared to a removable complete denture, can offer notable improvements in patients’ bite force [40], quality of life [41], and masticatory performance [42]. Our reports, given the results obtained both after one months and after one year, similarly indicate a clear improvement in all aspects investigated by the OHIP-14. Our study, therefore, follows what has been proven before; however, in our case, these measurements were repeated, therefore confirming that these improvements are stable over time, and proving that the benefits obtained with this kind of rehabilitation are not limited in time. Similarly, Bilhan et al. [43] have demonstrated a positive results for mandibular overdentures, with LOCATOR attachment providing the best results as defined by those patients, especially in situations where a reduced interarch space was present [44]. Few studies are available comparing other low-profile attachment; therefore, no comparisons can be traced between them.

The main difference in our paper is its longer follow-up, as patients were followed for two years after delivery, which is quite rare in the available literature [45,46]. Our article is the first to employ the OHIP-14 in an Italian speaking group of patients treated with two implant-supported mandibular overdenture.

Our study has many limitations; first and foremost, we used a completely subjective way of measuring retention, which is a major bias. Moreover, our sample is a particularly small one and our study was conducted in a private setting, as such, it included patients that can afford this rehabilitation in this kind of setting. More studies are needed to confirm our findings in order to compare two implant-supported overdentures to four implants, bar-retained, overdentures.

## 5. Conclusions

Edentulous patients can suffer a great impairment of their oral functions, causing a notable worsening of their eating capabilities and social well-being; removable dentures are a simple and relatively inexpensive treatment option, but, given their flaws, they cannot always provide an adequate treatment option. Given the results of our study, two implant-supported mandibular overdentures are an efficient way to rehabilitate edentulous patients, given their capability of improving these patients’ quality of life, and their positive outcome at a two year follow-up.

## Figures and Tables

**Table 1:** Characteristics of the sample.

Age	65.48 ± 5.23
Gender	17 (42.5%) Male, 23 (57.5%) Female

**Table 2:** Responses to the OHIP-14.

(Mean ± SD)	T0	T1	T2
OHIP-14	37.27 ± 3.49	20.27 ± 4.21 *	20.67 ± 3.89
Prostheses retention	\	2.22 ± 0.69 *	3.15 ± 0.89

* *p*-value < 0.05.

**Table 3:** Responses to each OHIP item.

(Mean ± SD)	T1	T2	T3
Q1	2.27 ± 0.87	1.3 ± 1.11	1.325 ± 0.99 *
Q2	2.15 ± 1.05	1.25 ± 0.95	1.275 ± 0.71 *
Q3	2.275 ± 0.87	1.45 ± 1.13	1.275 ± 0.75 *
Q4	3.425 ± 0.87	1.725 ± 1.32	1.65 ± 0.92 *
Q5	2.85 ± 0.97	1.5 ± 0.90	1.45 ± 0.81 *
Q6	2.15 ± 0.86	1.325 ± 0.82	1.375 ± 0.77 *
Q7	2.7 ± 1.04	1.925 ± 1.07	1.85 ± 0.86 *
Q8	2.85 ± 0.97	1.65 ± 1.00	1.625 ± 0.89 *
Q9	2.5 ± 1.08	1.375 ± 1.16	1.4 ± 0.95 *
Q10	2.75 ± 1.08	1.6 ± 1.12	1.575 ± 1.08 *
Q11	2.8 ± 1.04	1.175 ± 0.98	1.2 ± 0.99 *
Q12	2.675 ± 1.07	1.525 ± 1.15	1.45 ± 0.78 *
Q13	2.875 ± 0.96	1.55 ± 0.95	1.575 ± 0.87 *
Q14	3 ± 1.06	1.325 ± 0.91	1.275 ± 0.71 *

* *p*-value < 0.05.

**Table 4:** The retention offered by the mandibular IODs.

Absolute Frequency/Relative Frequency	T2	T3
Very high retention	6 (15%)	
High retention	19 (47.5%)	9 (22.5%)
Moderate retention	15 (37.5%)	20 (50%)
Weak retention		7 (17.5%)
Very weak retention		4 (10%)

## Data Availability

Data can be requested from the corresponding author.

## Appendix A. The Adopted Italian Questionnaire and Its English Translation

**ORAL HEALTH IMPACT PROFILE (OHIP-14)**
1. Ha avuto problemi nel pronunciare parole a causa di problemi con denti, bocca, o protesi?
2. Ha trovato che il suo senso del gusto fosse peggiorato a causa di problemi con denti, bocca, o protesi?
3. Ha avuto dolori fastidiosi in bocca?
4. Ha trovato difficile mangiare alcuni cibi a causa di problemi con denti, bocca, o protesi?
5. Si è sentito a disagio di fronte agli altri a causa di problemi con denti, bocca, o protesi?
6. Si è sentito teso o nervoso a causa di problemi con denti, bocca, o protesi?
7. La sua dieta è stata insoddisfacente a causa di problemi con denti, bocca, o protesi?
8. Ha dovuto interrompere i pasti a causa di problemi con denti, bocca, o protesi?
9. Ha trovato difficoltà a rilassarsi a causa di problemi con denti, bocca, o protesi?
10. Si è sentito un po’imbarazzato a causa di problemi con denti, bocca, o protesi?
11. Si è sentito un po’ irritabile con altre persone a causa di problemi con denti, bocca, o protesi?
12. Ha avuto difficoltà a fare i lavori usuali a causa di problemi con denti, bocca, o protesi?
13. Ha trovato che la vita in generale fosse meno soddisfacente a causa di problemi con denti, bocca, o protesi?
14. È stato totalmente incapace di agire o fare cose a causa di problemi con denti, bocca, o protesi?
**ORAL HEALTH IMPACT PROFILE (OHIP-14)**
1. Have you hade troubles pronouncing any word because of problems with your teeth, mouth or prostheses?
2. Have you felt that your sense of taste has worsened because of problems with your teeth, mouth or prostheses?
3. Have you had painful aching in your mouth?
4. Have you found it uncomfortable to eat any foods because of problems with your teeth, mouth or prostheses?
5. Have you been self-conscious because of problems with your teeth, mouth or prostheses?
6. Have you felt tense because of problems with your teeth, mouth or prostheses?
7. Has your diet been unsatisfactory because of problems with your teeth, mouth or prostheses?
8. Have you had to interrupt meals because of problems with your teeth, mouth or prostheses?
9. Have you found it difficult to relax because of problems with your teeth, mouth or prostheses?
10. Have you been a bit embarassed because of problems with your teeth, mouth or prostheses?
11. Have you been a bit irritable because of problems with your teeth, mouth or prostheses?
12. Have you had difficulty doing your usual jobs because of problems with your teeth, mouth or prostheses?
13. Have you felt that life in general was less satisfying because of problems with your teeth, mouth or prostheses?
14. Have you been totally unable to function because of problems with your teeth, mouth or prostheses?
